# Orofacial Trauma on the Anterior Zone of a Trumpet’s Player Maxilla: Concept of the Oral Rehabilitation—A Case Report

**DOI:** 10.3390/ijerph17249423

**Published:** 2020-12-16

**Authors:** Miguel Pais Clemente, André Moreira, Nádia Carvalho, Gilberto Bernardes, Afonso Pinhão Ferreira, José Manuel Amarante, Joaquim Mendes

**Affiliations:** 1 Departmento de Cirurgia e Fisiologia, Faculdade de Medicina, Universidade do Porto, 4200-319 Porto, Portugal; amarante@med.up.pt; 2 Labiomep, INEGI, 4200-465 Porto, Portugal; jgabriel@fe.up.pt; 3 Oral Rehabilitation, Faculdade de Medicina Dentária, Universidade do Porto, 4200-135 Porto, Portugal; andre.luis.sa.moreira@gmail.com; 4 INESC TEC, Faculdade de Engenharia, Universidade do Porto, 4200-465 Porto, Portugal; n.svc@fe.up.pt (N.C.); gba@fe.up.pt (G.B.); 5 Faculdade de Medicina Dentária, Universidade do Porto, 4200-135 Porto, Portugal; aferreira@fmd.up.pt; 6 Faculdade de Engenharia, Universidade do Porto, 4200-465 Porto, Portugal

**Keywords:** orofacial trauma, orofacial pain, wind instrument player, oral rehabilitation, embouchure mechanism, sound quality, orthodontics, intra-oral appliances

## Abstract

Background: The occurrence of an orofacial trauma can originate health, social, economic and professional problems. A 13-year boy suffered the avulsion of tooth 11 and 21, lost at the scenario. Methods: Three intraoral appliances were manufactured: A Hawley appliance with a central expansion screw and two central incisors (1), trumpet edentulous anterior tooth appliance (2) and a customized splint (3) were designed as part of the rehabilitation procedure. Objectively assessing the sound quality of the trumpet player with these new devices in terms of its spectral, temporal, and spectro-temporal audio properties. A linear frequency response microphone was adopted for precision measurement of pitch, loudness, and timbre descriptors. Results: Pitch deviations may result from the different intra-oral appliances due to the alteration of the mouth cavity, respectively, the area occupied and modification/interaction with the anatomy. This investigation supports the findings that the intra-oral appliance which occupies less volume is the best solution in terms of sound quality. Conclusions: Young wind instrumentalists should have dental impressions of their teeth made, so their dentist has the most reliable anatomy of the natural teeth in case of an orofacial trauma. Likewise, the registration of their sound quality should be done regularly to have standard parameters for comparison.

## 1. Introduction

The occurrence of an orofacial trauma can originate health, social, economic, and professional problems. Many factors can be associated to the event that can lead to an orofacial trauma; however, the age of the individual is intimately related to specific episodes. It is well known that children, particularly between 10–14 years old, can practice risky sports which may originate an orofacial trauma [1,2,3,4]. The consequences of these events are unpredictable since the clinical findings can be dispersed, from a simple tooth fracture involving the enamel to a tooth avulsion [5], or a fracture of the zygomatic or malar bone, for example.

The severity of an orofacial trauma can require a multidisciplinary approach on treatment procedures that usually are associated with orofacial pain. The complementary actions of distinct health professionals, such as dentists [6], and maxillofacial [7] and otorhinolaryngology surgeons, are quite frequent. The specificity of certain medical specialties can enforce the success of the treatment plan outcome [8,9]. Understanding the interaction of these different areas can be useful, on a rational evaluation of the patient and in establishing a diagnose. For example, a child that has an overjet of the upper central incisors can have a higher chance to fracture the clinical crown after a fall [10]. Independently to the fact that the tooth fracture can reach the tooth pulp, where an endodontic treatment is needed [11,12], it is worth noticing that the dental restoration technique can, unfortunately, be insufficient to avoid the restoration to break due to the applied forces when the patient is biting with the anterior teeth. This is also extremely important and relevant, for example, to the phonation of a child, where sounds involve the tongue tip making contact with the upper teeth to form a constriction. These aspects involving the articulation and sound producing during the speech process can be easily affected after an orofacial trauma. Sometimes the need of a speech therapist can be necessary.

Some of the above mentioned considerations can have practical consequences after the occurrence of an orofacial trauma when playing on the courtyard at school with some friends [13], practicing sports, or even at a recreational activity such as riding a bicycle. At an older age, when an individual reaches the early stage of adulthood, the orofacial trauma is usually associated with motor vehicles accidents, the practice of combat sports, or violence episodes [14]. It is possible to prevent or reduce the damage of specific orofacial structures with the use of sports mouthguards that are typically used in sports such as rugby, field hockey, handball, basketball, or water polo. In that case, it is still scarce to use them during sports practice [15,16,17,18,19] and even less at leisure activities. However, these ludic moments can bring unexpected complications that may result in orofacial trauma [20].

This research intends to highlight a clinical case of a 13-year boy who had an accident while riding his bicycle. As a result, the child suffered an orofacial trauma, with the avulsion of the two maxillary central incisors, tooth 11 and 21. In such an emergency case, the reimplantation within 30 min is the best treatment option [21,22]. The treatment of an anterior dental trauma can result in multiple interventions over the patient’s lifetime [23]. Independently to the fact that this clinical situation is a severe drama, to any individual, this has the aggravating factor of being a wind instrument player. The patient studies at a musical school and, like other members of his family, intends to continue to study and perform in this field. Therefore, this orofacial trauma has a significant impact on his musical activity since the teeth that sustained the upper lip, while doing the embouchure, were lost. The question is, what is the proposed treatment concept of the oral rehabilitation procedure for a thirteen-year boy, who is a musician, or intends to become a professional brass instrument player and has had this type or orofacial trauma?

A trumpet player, besides the pulmonary activity and the respiratory system functions, has a fundamental orofacial activity during the embouchure mechanism that will allow the instrumental sound production. For this purpose, it is relevant the placement and position adopted by the mandible regarding the upper jaw with the inherent biomechanics of the temporomandibular joint, the position of the tongue, the muscular activity of the orbicularis oris, the masticatory muscles and finally the fundamental contribution of the anterior teeth, of the upper and lower jaw, but in particular, the upper central incisors, since there is an important support of the mouthpiece on the upper lip that likewise induces pressure on teeth 11 and 21. In the past, other studies have introduced exciting information regarding the induced pressure that a brass instrument player can apply towards the orofacial structures, namely, the upper and lower teeth. This was done with the piezoresistive sensors providing information that a trumpet player can reach a force of 172 gf, for example [24]. The application of sensors can be interesting from a clinical point of view to monitor the gesture technique of the cranio–cervico–mandibular complex of wind or string instrument players [25,26,27]. This information helps the dentist to understand their complaints better when attending a dental office seeking a solution for orofacial pain, or temporomandibular disorders.

The contribution of new techniques seeks to complement theoretical information which musical teachers understand, about which medical doctors eventually read or learn and join the information together. The information can then be used in practice to study and analyze a wind instrument player from the embouchure point of view with a particular focus of the embouchure mechanism [28] with the orofacial structures involved, and where the sound production obtained, is one of the main objectives of this research. Combining the field of performing arts, engineering, and performing arts dentistry will be reported throughout this paper, with the intention of describing the best treatment taking into consideration the orofacial trauma of the trumpet player.

## 2. Materials and Methods

The subject gave the informed consent before participating in the study. This investigation was made in accordance with the revised Helsinki Declaration (1983).

### 2.1. Case Preparation

**Figure 1:**
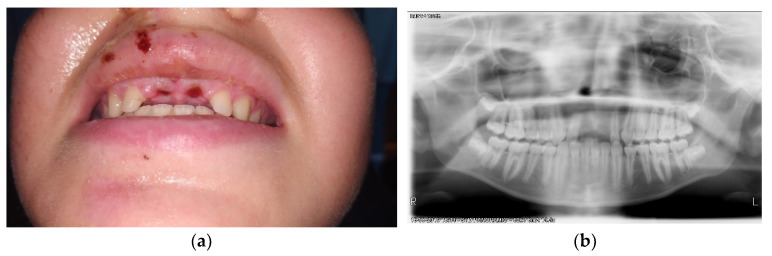
(**a**) Orofacial trauma with upper central incisors missing; (**b**) Panoramic X-ray confirming avulsion of teeth 11 and 21.

**Figure 2:**
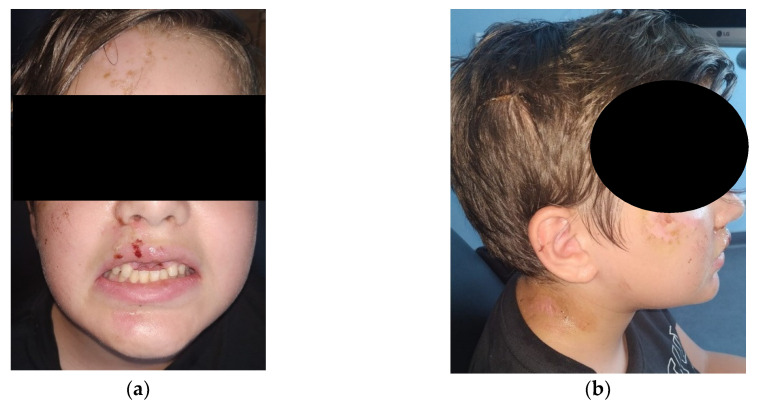
(**a**) Orofacial trauma with upper lip laceration; (**b**) Extension of lesions on the cranio–cervico–mandibular complex.

A 13-year-old boy was referred to a dental clinic specialized on the attendance of wind and string instrument players. After a complete anamnesis, it was possible to know that the patient suffered an accident while riding his bike, with direct implications on the orofacial tissues, since there was the avulsion of the upper central definitive teeth, tooth 11 and 21, that were lost at the accident scene. An exhaustive clinical examination was performed with an extra-oral palpation on the masticatory muscles and the temporomandibular joint (TMJ), where it was possible to observe a discrete discomfort on both TMJ. In the intra-oral physical examination, it was possible to see the dental alveolus of the missing teeth, 11 and 21 (Figure 1a) and in the panoramic radiographic examination (Figure 1b), it was possible to verify on the maxilla the absence of the two central incisors. The extra-oral photographs are elucidative of the extent of the lesions that occurred in the sequence of this orofacial trauma (Figure 2).

**Figure 3:**
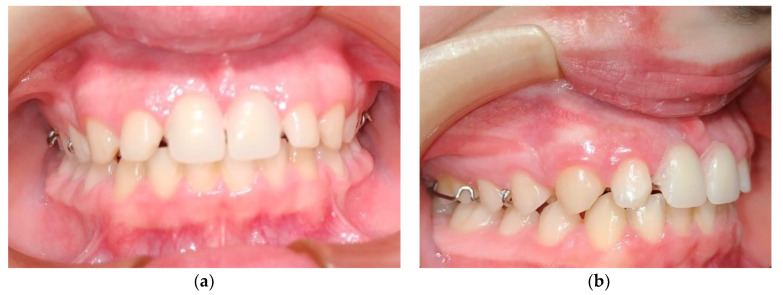
(**a**) Intra-oral view of the Hawley appliance with two central incisors; (**b**) Lateral right view of the occlusion of the trumpet player and the appliance.

At this point, it was established as a diagnostic hypothesis the oral rehabilitation with the inherent implications of the maxillary and mandibular growth. It is worthy to highlight that the replacement of the missing tooth should only be done as a definitive approach at the adult age, so a Hawley appliance with an expansion screw was fabricated with two central incisors. For this purpose, a dental impression of the upper and lower jaw was made to provide dental casts to study the best design of the appliance mentioned above and other solutions that could be beneficial for the trumpet player (Figure 3).

**Figure 4:**
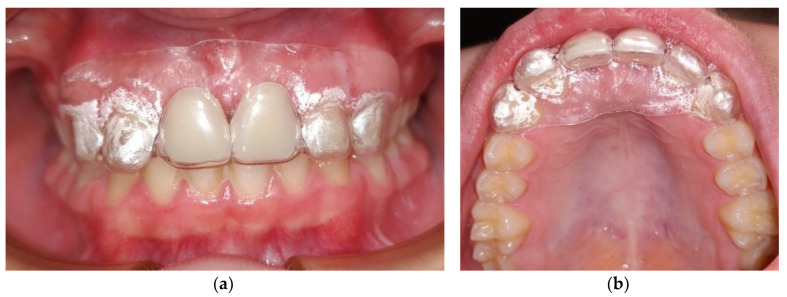
Trumpet edentulous anterior tooth appliance: (**a**) Front view; (**b**) Palatal view with the EVA material design and retention on the adjacent teeth.

**Figure 5:**
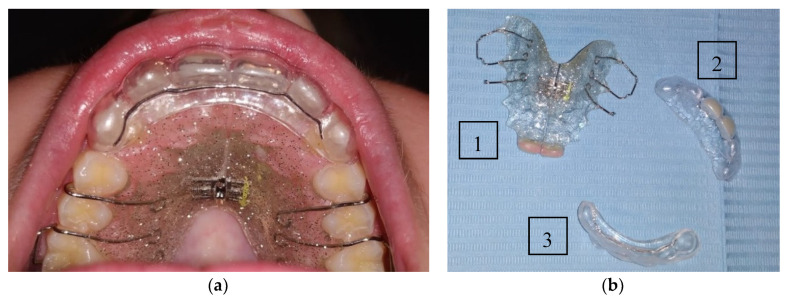
The customized splint placed over the Hawley appliance: (**a**) appliance in place; (**b**) The three intra-oral devices fabricated for this clinical case, the Hawley appliance with central expansion screw and two central incisors (1), the Trumpet edentulous anterior tooth appliance—TEATA (2), the customized splint (3).

A new appliance was manufactured with a 1 mm EVA foil from Erkodent, using the Erkoform 3D-motion vacuum machine, Figure 4. This appliance was denominated the trumpet edentulous anterior tooth appliance (TEATA) since it has the two missing central incisors. This intra-oral device intends to provide an alternative to the Hawley appliance when playing the trumpet. However, another appliance was still made to offer more stability of the anterior teeth, Figure 5. This was a vacuum formed retainer of 1 mm foil, also made of EVA, to place on top of the Hawley appliance, with its design extending from canine to canine, tooth 13 to 23.

### 2.2. Data Collection of the Trumpet Sound Quality

**Figure 6:**
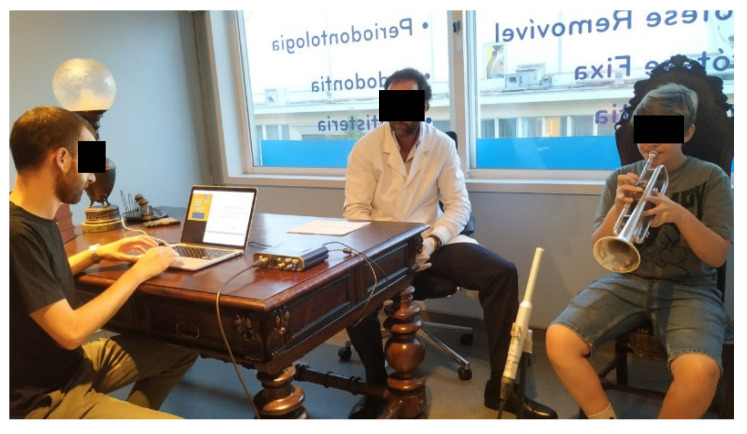
Capture settings of the trumpet sound quality using low-level audio descriptors.

The design of this research was divided in two parts. The first part involves general considerations that should be taken into account within a dental appointment of a trumpet player with an orofacial trauma. The second part concerns the data collection of trumpet sound quality. Four recording sessions were conducted over ten months (10 September 2019; 14 October 2019; 3 February 2020; and 3 June 2020). The patient performance was recorded using the three intra-oral appliances conditions in all sessions, testing the effect of these devices on the trumpet sound quality during a musical performance (see Figure 6). Due to the patient’s limited pitch range without excessive effort, we consider the C major arpeggio (C, E, and G) in the octave 3 to be representative of different register conditions.

The Behringer ECM8000 linear frequency range response microphone was adopted for precision measurement. Recording conditions were replicated in the four sessions. The measurement microphone was placed about one meter facing the trumpet bell. The spatial locations of the patient and the recording devices were similar across sessions to minimize the impact of the room acoustics. The order in which the three intra-oral devices were recorded varied in each session to reduce the possible effect of playing fatigue. Each pair of register versus intra-oral devices were repeated three times in each session.

### 2.3. Feature Extraction and Selection

Audio descriptors characterize an excerpt of an audio signal in terms of its spectral, temporal, and spectro-temporal properties. Audio descriptors are divided into time-varying descriptors and global descriptors [29]. The former provides a morphological description of the analyzed sound by computing indicators per sequential window audio frames at a time, thus resulting in a time series—i.e., a temporal sequence of values. In our work, we adopt standard values of 2048 samples for window size and 512 samples of overlap, considering an audio sample rate of 22,500 Hz. The former summarizes the time-varying descriptor sequence to single values per analyzed sound. To this end, descriptive statistics are typically adopted, such as minimum or maximum values, the mean or median, and the standard deviation or interquartile range (IQR), i.e., the difference between the 75th and 25th percentiles of the sequence of values [30]. We adopt the median and IQR as descriptive statistics as it is resistant to possible outliers. The librosa library [31] was adopted to compute the audio descriptions.

From a technical perspective, the sound quality results from numerous acoustic properties, including pitch, loudness, and timbre content [32,33,34]. The scope of automatic performance can be split into two main categories: (1) score-independent and (2) score-dependent descriptors [35]. Score-independent descriptors are derived from audio signals without using additional information about the musical score being performed. Representative works on score-independent features are Abeßer et al. [36], Han and Lee [37], and Wu et al. [38]. Score-dependent descriptors are derived by leveraging the information provided by the score being performed, such as note and rhythmic accuracy. For work on score-dependent features, please refer to Vidwans et al. [35]; Devaney, Mandel, and Fujinaga [39]; and Mayor, Bonada, and Loscos [40].

The list of audio descriptors adopted in our study is shown in Table 1. It includes score-independent descriptors only, following earlier proposals by Vidwans et al. [35] and Wu et al. [38].

## 3. Results

For each audio descriptor, we calculate (1) time-varying descriptors, (2) global descriptor statistics for the three intra-oral appliances per register, and (3) global descriptors statistics per intra-oral appliance (merging all register values).

**Figure 7:**
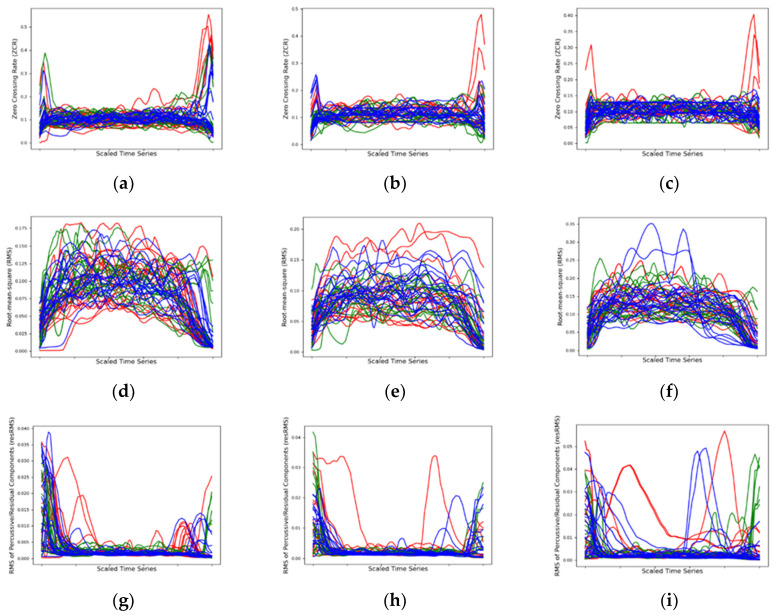

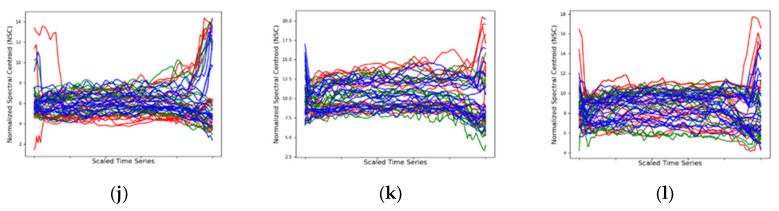
Time-varying descriptions registers for ZCR ((**a**) low, (**b**) mid, (**c**) high); RMS ((**d**) low, (**e**) mid, (**f**) high); resRMS ((**g**) low, (**h**) mid, (**i**) high); NSC ((**j**) low, (**k**) mid, (**l**) high). Red Line—Hawley appliance central incisors (HACI), Green Line—TEATA, Blue Line—Customized Splint. The time series of all plotted sounds were scaled on the x-axis to uniform duration.

Time-varying descriptors are shown in the line-graphic plots of Figure 7. For each descriptor, it is shown three line-graphics detailing the time-varying morphology of all recorded sounds per register. The temporal dimension of all sounds (ranging from 1 to 2 s) is aligned to a unitless axis, which stretches all curves to the same start and end positions. In the resulting representation, the time-varying descriptor behavior on important structural moments of the sound, such as the attack, sustain and release, can be better compared.

**Figure 8:**
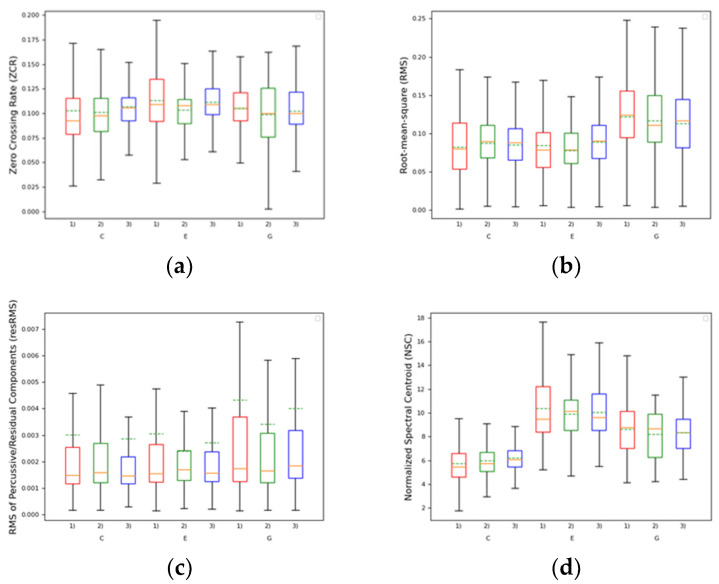
Box plots of global statistics per register and intra-oral appliances for (**a**) ZCR; (**b**) RMS; (**c**) resRMS; and (**d**) NSC. Numbers in the x-axis denote intra-oral appliances: (1) HACI, (2) TEATA, and (3) Customized Splint. Low, mid, and high registers are expressed by the performed note (i.e., C, E, and G, respectively). Orange and dashed green lines denote median and mean values, respectively.

Global descriptor statistics for the three intra-oral appliances per register are shown in the boxplots of Figure 8, We denote register conditions by their corresponding note: C, E, and G. Intra-oral appliance conditions are numbered according to the following order: (1) Hawley appliance with an expansion screw was fabricated with two central incisors (HACI), (2) trumpet edentulous anterior tooth appliance (TEATA), and (3) customized splint. The box ranges from the lower (Q1) to upper (Q3) quartiles (being equal to the 25th and 75th percentiles). The IQR, a measure of statistical dispersion or variability, is computed as the difference between Q3 and Q1. The second quartile Q2 equals the median of the descriptor data and is represented in the boxplot as an (orange) line crossing the box. Lower and higher whiskers are computed as Q1−1.5 IQR and Q3+1.5 IQR, respectively. Outliers in descriptor data fall outside the whisker’s range.

Global descriptors statistics results per intra-oral appliance, merging all register values, are shown in Table 2. We apply the non-parametric Friedman test of differences across repeated measures of the three intra-oral appliance conditions per descriptor to determine whether there is a statistically significant difference (*p*-value) across the appliances. Results are shown in Table 3.

### 3.1. Pitch

The time-varying descriptions in Figure 7a–c denote overall stability in the zero-crossing rate (ZCR) rate of the trumpet sound, namely during the sustain part of the trumpet note envelope. The trumpet envelope note’s attack and release have higher variance due most probably to typical noisy transients. To minimize transient noise data in the analysis, we adopt median and IQR analysis.

The global statistics in Figure 8a and Table 2 for the zero-crossing rate descriptor show that pitch fluctuations are observed in the collected data. No clear tendency can be observed across the three intra-oral appliances, as the pitch inflexions in Figure 8a differ per register. However, when comparing the intra-oral appliances with merged data across registers Table 2, statistically significant inflexions can be observed across the median values (*p*-value < 0.001). The median values for the HACI (0.01) are followed by the TEATA (0.103) and, finally, the customized splint (0.106).

### 3.2. Loudness

An expected increase in the root mean square (RMS) descriptor from low to higher registers is verified in the collected data due to the higher pressure needed by higher trumpet registers. These results are expressed in both the time-varying descriptions in Figure 7d–f and the boxplot in Figure 8b, whose mean (dotted green line) and median (orange line) for the upper register sounds have higher RMS values. In Table 2, the RMS’s IQR shows the overall stability per intra-oral appliance. The TEATA (0.047) presents a lower IQR, thus denoting higher stability in the loudness of the trumpet sound quality. The customized splint (0.523) and the HACI (0.067) perform worse in terms of loudness stability.

The root mean square of the percussive/residual components (resRMS) exhibits the same tendency as the RMS descriptor above with higher values in the trumpet’s upper register. Furthermore, in the time-varying descriptions, we observe that the percussive/residual components exist mostly in the attack and release phases of the trumpet note envelope. Conversely to all remaining descriptors, where median values excluding outliers from the data are somewhat more robust and informative of the quasi-stationary (i.e., steady) quality components of the sound, the transient nature of the percussive/residual components is better captured by the mean the global statistics shown in Figure 8c and Table 2. In comparing the means per intra-oral appliance in Figure 8c the same tendency is found for all trumpet registers. Table 2 further enforces this tendency. Despite the weaker statistical significance (*p*-value = 0.058), the data suggests the presence of much lesser percussive/residual components in the TEATA (0.0028) in relation to the customized splint (0.0035) and the HACI (0.0033).

### 3.3. Timbre

The time-varying descriptions in Figure 7j–l denote overall stability in the normalized spectral centroid (NCS) of the trumpet sound, namely during the sustain part of the trumpet note envelope. The trumpet envelope note’s release has noticeably higher variance due most probably to typical noisy transients. In the low register, the TEATA and the customized splint shows an expressive increase in the normalized spectral centroid (NSC). In the mid register, only the TEATA has a significant increase in terms of NSC. In the high register, no expressive differences exist. Higher NSC values correlate with higher auditory brightness, which is associated with better sound quality in brass instruments [42]. The global statistics in Table 2 do not provide much evidence on NSC when aggregating all register data per intra-oral appliance.

## 4. Discussion

Orofacial trauma of the anterior maxilla has in the tooth avulsion one of the principal emergency that can occur at a dental office where specific guidelines and recommendation should be followed [43,44]. There are fundamental issues to have in mind, such as tooth replantation, extra-oral time tooth is maintained before replantation, transportation method of the tooth, preservation of the periodontal ligament, maintenance of the root surface the most intact as possible, endodontic treatment if the tooth is immature or not, stabilization process of the replanted tooth, and antibiotics therapeutics.

Understanding the differences in the avulsion procedure of primary or permanent teeth is fundamental since a primary tooth is never replaced if avulsed [45]. It is important to highlight the guidelines for the traumatic dental injuries of the avulsed teeth, namely, the avulsion of permanent teeth [43]. Fouad et al. developed a consensus statement after an update of the dental literature and discussions among expert groups, stating among many other things that replantation is, in most situations, the treatment of choice but cannot always be carried out immediately [43]. Appropriate emergency management and a treatment plan are important for a good prognosis [43]. Crown fractures and luxation are the most commonly occurring traumatic dental injuries of permanent teeth that frequently occur in children and young adults [46].

Timely treatment of injured anterior teeth prevents much further damage and expensive treatment for the affected young patients [23]. Brüllmann et al. stated that avulsed frontal teeth often cannot be saved because of improper or lack of initial treatment, and the result is the need of multiple interventions over the patient’s lifetime, which also carry a high financial cost. This is similar to what happened in this case. Therefore, when this accident occurred, the patient was running back to his house to report what happened to his parents. Immediately, there was the awareness of the importance in recovering his lost tooth, so the boy and his parents went back to the scenario where the accident occurred to try and recover his teeth. Independently to the fact that in this particular case the teeth was not found by the parents at the location where the young boy suffered the bicycle accident, it is extremely important to have this perception that an avulsed tooth can be implanted. Not replanting the tooth is an irreversible decision and therefore saving it should be attempted [43].

This orofacial trauma was part of recreational activities that were associated with an increased risk for sports-related injuries [47]. In the dental appointment, it was referred by the patient’s parents that they intended to bring the teeth in “a box with milk”. Hiremath et al., referred that although a balanced salt solution is the reference for storage media of an avulsed teeth, an orofacial trauma can occur at a place where this solution is not available easily, and therefore, for example, in a tropical country it is suggested coconut water for this purpose since it is the natural, isotonic beverage that is readily accepted by the body because of its sterility, pH, and electrolytic balance. Pohl et al., describe that physiologic storage of avulsed teeth in one of the major issues for a good prognosis of the dental treatment [48]. The information about the way to keep an avulsed tooth can differ, for example, between school nurse teachers compared with parents, Kinoshita et al., reported different manners in which avulsed tooth was handled at home versus at school [48]. Often teeth avulsed at school were kept under wet conditions being the time until their replantation ranged between 0.5 and 3.5 h, while the avulsed teeth that occurred at home or near, were left under dry conditions and their time until replantation ranged scattered between 0.5 and 12 h [48]. Having the knowledge of how to lead in a case of orofacial trauma, is essential. Von Brünen et al. carried a survey assess the management of avulsions in Switzerland as the common treatment procedures used by Swiss dentists in such cases, with a response rate of 41% (n = 1350), where seventy-eight percent (78.1%) of the respondents had received postgraduate dental trauma education. On average, two avulsions per practitioner had been seen in the past three years and (81.1%) of the respondents mentioned they had a tooth rescue box in their office [49]. However, the literature indicates that sometimes the level of information is not always homogeneous, between healthcare workers, dental students, parents or caretakers, being necessary the implementation of educational programs with public health policies to manage orofacial trauma, in this particular issue, avulsion cases [50,51,52,53,54,55].

In the present case, it was imperative to decide the rehabilitation process of the avulsed tooth 11 and 12 in a manner that did not compromise the craniofacial growth of the patient and would allow this young musician to feel secure in the stability that his new teeth would provide to the embouchure mechanism while playing the trumpet. The adopted solution was the fabrication of a Hawley appliance with a central expansion crew and with the placement of tooth central incisors (HACI—Hawley appliance central incisors). The purpose of this appliance, besides the functional and aesthetic rehabilitation of the missing teeth, was to follow the transverse skeletal and dentoalveolar modifications during the growth of the boy. Marshall et al., when analyzing 36 untreated subjects with Class I occlusion at approximate ages of 7.5, 10.3, 12.9, 16.5, and 26.4 found out that maxillary first and second molars upright lingual by 3.3° and 5.9°, respectively, while maxillary first and second intermolar width increased by 2.8 and 2.0 mm, respectively [56]. So, the evolution of the changes in molar crown torque and intermolar arch width of permanent first molar eruption to early adulthood of the young trumpet player could be accompanied by any necessary adjustment of the central expansion crew of the HACI, where an intentional diastema was implemented in order to make it viable, for the eventual expansion of the HACI. As a result, the management of this case intends to monitor the craniofacial morphology during the growth of the musician besides this provisional solution of oral rehabilitation. Hesby et al. performed thirteen maxillary and mandibular transverse measurements using casts and posteroanterior radiographs from 36 Class I untreated subjects, from approximate ages of seven and half years to 26 and half years, verifying that there is a pattern of width changes in the maxilla, the maxillary alveolar process, the maxillary first molars, the mandibular first molars, and the mandibular alveolar process that occurs as a gradient in the vertical dimension [57]. Maxillar molars erupt with buccal crown torque and upright with age, accompanied by concurrent increases in maxillary and mandibular intermolar widths [56,57].

Therefore, the objective of our study was to restore the capacity of the child to play his wind instrument and follow his growth pattern regarding the orofacial structures. An understanding of the timing, magnitude and direction of facial growth enables orthodontists to plan the treatment of skeletal discrepancies in an attempt to achieve a more stable and pleasing result [58]. Thordarson et al. found out that the most marked out changes in craniofacial morphology from 6 to 16 years, in 182 Icelandic children, is related to the mandibular prognathism, the mandibular plane angle and the inclination of the lower incisors [58]. Trunetouth et al. stated that the growth of the cranium largely ceased by nine-years indicating precocious growth of the brain in relation to the face [59]. The size of the face increased relative to the cranium by 69%. The lower face increased relative to the upper face by 22%. Failure of such a mechanism could play an important role in the etiology of malocclusion and its treatment by functional jaw orthopedics [59]. Similarly, Arboleda et al. evaluated the craniofacial growth of Colombian mestizos. Four age cohorts, including a total of 458 children and adolescents (262 males and 216 females), were included in this mixed-longitudinal study. The cohorts were first measured at ages 6, 9, 12, and 15 and every year thereafter for three years [60]. Arboleda et al. results allowed to verify the following: the cranium grew less than the craniofacial, which in turn grew less than the facial dimensions. In addition, vertical dimensions showed more growth than anteroposterior dimensions, which in turn grew more than transverse dimensions [60]. Therefore, the long-term prognosis of the transverse growth of the maxilla of our trumpet player can be favorable with no major changes expected, especially if it is taken in consideration that the patient has a stabilized occlusion, with a Class I angle molar and canine relationship and no posterior crossbite. However, the use of an orthodontic appliance with two anterior teeth can present more advantages to use by a young patient, than a “standard” removable prosthesis with the two central incisors. These factors can also make a difference when we are talking of a delicate age, in terms of personality and development, when usually close friends and family want to know more about the clinical situation after an orofacial trauma like this one.

On the other hand, as clinicians and researchers, the authors of this paper understood the delicate situation that needed to be solved regarding the extra volume the trumpet player now had in his mouth with the HACI. To a musician, any kind of change in the orofacial structures can be detrimental to the embouchure mechanism and likewise his ability to play. In this particular situation, it was the musician’s capacity to learn again the way to position the tongue, the lips and mouthpiece in order to be able to perform a musical note. The position of the two central incisors was not a problem since his previous natural teeth 11 and 21 were slightly crowded, and now the HACI teeth were placed aligned. The lips would adjust to the more orthopositioned incisors, as the teeth before being avulsed presented a protrusion. The question was how the airflow could be sufficiently smooth in order to produce good sound quality? How was the tongue of the trumpet player able to adapt to this new intra-oral condition? Because it is well known that any children that use these apparatuses have difficulty in speech articulation [61,62]. Chen et al. [63], after a systematic review of the literature, summarized the associated problems of speech and intra-oral appliances, being possible to verify that these can lead to speech difficulties. Lingual fixed appliances, palatal expanders, and Hawley retainers have an evident influence on speech production. The /i/, /s/, /t/, and /d/ sounds are the primarily affected ones [63].

The need to respond positively to the above-mentioned questions, made with which a multidisciplinary team gather around this clinical situation, in order to provide solid answers and quantify the new embouchure in terms of sound quality. In the meanwhile, other two prosthetic solutions were provided to the trumpet player. The first was an appliance that should be placed on top the HACI in order to promote a higher stabilization while the musician was playing his instrument. This appliance was like a custom-made splint from the upper left maxillary canine extended till the right maxillary canine. This custom-made splint was made from an EVA sheet foil of 1 mm and had a titanium arch wire in the palatal surface in order to allow the dissipation of forces produced by the trumpet player. This would provide a higher confidence on the musician when applying his mouthpiece against the lip, and a higher resistance to the existing pressures, once the two central incisors of the HACI by themselves alone only had support on the vestibular and palatal crest of the anterior maxilla. The second solution was denominated as the trumpet edentulous anterior tooth appliance (TEATA) and the main goal of this apparatus was to prepare a solution for the young musician in case of any kind of maladjustment on his musical performance when playing the trumpet with the HACI, or the customized splint. Fortunately, there was a solution and a treatment option that included these prosthetic appliances like the TEATA for the anterior missing teeth. Our treatment plan intended to provide an alternative, where the TEATA seemed a solution with an optimal aesthetic appearance and occlusal function offering a high probability of an overall favorable prognosis. However, would it work in terms of stability and sound quality during a musical performance? The design of the TEATA was made to adapt and gain retention on the adjacent tooth of the upper central incisors, the lateral incisors and the canine, in order to gain a higher anchorage. The patient positively accepted this device during the dental consultation, and the HACI apparatus was immediately placed aside to a second plan since the comfort, retention, and occupying space within the mouth was much more favorable with the TEATA appliance. Although the patient subjectively mentioned these features, it was now time to quantify the impact of these intra-oral devices objectively.

The assessment of instrumental performance is subjective to a level that multiple professional musicians typically discuss: the search for a unique sound or interpretation. While no two musicians have the same, or even similar, sound ideals [64] and the standard for sound quality varies, joint problems, such as a shrill piercing quality in the upper register and a fuzzy and unclear sound in the lower register, have been identified [64]. Numerous studies have attempted to determine timbre components that discriminate instruments and sounds [65,66,67]. While fewer studies have examined the attributes contributing to judgments of sound performance quality. However, the automatic assessment of music performance has become an area of increasing interest due to the growing number of technology-enhanced music-learning systems. There are studies that provide an objective assessment of instrumental performance, with a focus primarily on pitch and rhythmic (onset) accuracy [35,38,68]. Other researches address different but also essential aspects of performance, such as sound quality or timbre, which equally play a significant role in assessing musical performances [69,70]. For a comprehensive survey on the objective assessment of instrumental performance please refer to Lerch et al. [71]. However, obtaining objective or quantifiable criteria for the assessment of timbre quality is challenging. Objective descriptors, also referred to as audio descriptors, are indicators extracted from the audio signal. It typically quantifies in value (or a set of values) various characteristics of an audio signal.

In characterizing of trumpet sound quality, Madsen and Geringer [72] examined preferences for good/bad sound quality in trumpet performance. Though the two qualities were audibly distinguishable when presented without accompaniment, the only difference their published analysis discussed was the amplitude of the second fundamental. In a different study, an equalizer was used to amplify or dampen the third through eleventh harmonics of recorded sounds to be rated in quality [42]. For the brass instruments notes, a darker sound, caused by dampened harmonics, was judged to have a lower quality than the standard or brightened conditions. Knight et al. [69] studied sound quality in brass instrument performance based on subjective ratings of good/bad timbre among sounds with the same pitch and loudness played by the same instrument. Support vector machines (SVM) were used to discriminate good and bad sounds based on different score thresholds and groupings. Picas et al. (2015) [70] studied the overall goodness of flute, clarinet, and trumpet sounds. The quality of a performed sound was defined based on its dynamic pitch and timbre stability, timbre richness, and attack clarity. Based on recordings of good and bad examples of each of the aforementioned sound qualities, machine learning models were obtained to classify performed sounds in real-time.

The objective analysis of sound quality used in this study, regarding the pitch steadiness expressed by smaller IQR values demonstrates that the customized splint performs better (0.027), followed by the TEATA (0.035), and, finally, the HACI (0.038). Regarding the loudness, the TEATA presented a lower IQR (0.047), which denotes higher stability in the loudness of the trumpet sound quality. The customized splint covering the HACI (0.523) and HACI (0.067) perform worse in terms of loudness stability. This is in accordance with the initial subjective evaluation of the trumpet player that immediately said he would play with the TEATA since it occupied less space inside his mouth. It is interesting to notice that the values associated to loudness stability validate the musician opinion. However, the HACI still present an expressive IQR difference when compared to the TEATA, (0.067) to (0.047), respectively, which means that, with time, the musician could start to adapt himself to the palatal surface of the HACI.

The timbre can be characterized in the low register, where the TEATA and the customized splint shows an expressive increase in normalized spectral centroid terms. In the mid register, only the TEATA have a significant increase in terms of the normalized spectral centroid. In the high register, no expressive differences exist. Higher normalized spectral centroid values correlate with higher auditory brightness, which is associated with better sound quality in brass instruments [42]. These results are consistent with the initial hypothesis concerning the fact that the TEATA seemed a promising solution for the young trumpet player, in terms of stability and sound quality. It was notorious the benefits of being able to develop the TEATA, that occupies less space inside the oral cavity allowing the musician to be comfortable during his embouchure mechanism and musical performance. However, the advantages shown by the objective analysis of sound quality in this study have to be looked with caution, since there can be side effects being caused to the adjacent natural teeth, the lateral incisors to be more specific, once the applied forces on the lip by the trumpet mouthpiece are being transmitted to tooth 12 and 22. Radiographic control of the lateral incisors and test vitality should be done at routine dental appointments throughout the whole process, till the adult age, where the final restorative procedure will be made.

Providing a rational evaluation and a proposed treatment concept of oral rehabilitation after an orofacial trauma like the one described in our work obliges to consider the anterior zone of the maxilla as an esthetic zone where, implants, abutments and eventually biomaterials for guided bone regeneration shall be taken in consideration. Mahn et al. describe some of these issues where the esthetic replacement of a maxillary central incisor using a dental implant can be a challenging task [73]. Advances in dental materials have led to the introduction of zirconia abutments and crowns that can be synergistically combined with other ceramic materials [73]. A patient’s aesthetic expectations must also be evaluated together with their lip activity and lip length. In this particular case, the lip function is extremely relevant since the patient is a brass player. A high smile line poses considerable challenges when planning for implant-supported restorations in the aesthetic zone because the restoration and gingival tissues are completely displayed [74]. After a tooth avulsion bone remodeling will inevitably occur. It should be expected a certain amount of bone resorption horizontally (buccolingual) as well vertically (apicocoronal) that can be justified due to the trauma, but also by the physiological law of bone maintenance which requires a certain daily stress/strain stimulus [75,76]. The stability and health of the peri-implant hard and soft tissues are necessary for success and long-term maintenance of dental implants. Beyond hard tissues augmentation, also soft tissues should be treated to offer a good peri-implant sealing at the new biological space (from the free gingival margin to the implant neck). Soft tissue thickness can be increased by modifying either bone or tissues. Puisys and Linkevicius reports different methods for soft tissue augmentation, such as flattening of the alveolar ridge, subcrestal implant placement, tent-pole technique, and vertical-horizontal augmentation, with various grafting techniques [77]. Seven variables described by Furhauser et al. may enhance the pink esthetic score by modifying the critical and subcritical contour of the abutment–crown complex (gingival margin level, interdental papillae, gingival contour, alveolar process, gingiva color, and texture) [78]. The creation of a provisional restoration prior the fabrication of the definitive restoration is recommended, which will serve for duplication of the correct emergence profile [79]. For the final restoration a zirconia abutment could be the choice for this case because its excellent biocompatibility and good aesthetical appearance, especially when the patient has thin gingival biotype. However, the fact that values of fracture strength are not as good as conventional titanium abutments should be considered [80]. To offer a fixed prosthetic solution two implant-supported crowns should be the first choice, avoiding the preparation of the upper canines and upper lateral incisors to support a six-element tooth-supported bridge. The replacement of missing teeth using implant-supported restorations is a well-accepted treatment approach [81,82]. Regarding the patient’s age, the oral rehabilitation treatment with implants should be postponed till his adolescence is completed [83].

In the presence of a juvenile wind instrument player that suffered the avulsion of two central incisors, which were lost on the accident therefore not being able to be replanted, making a decision about the following steps is a sensitive procedure. Decisions regarding cranial growth, and surgical and prosthetic treatment have to be necessarily made in conjunction with the patient and his parents. In our particular case, this had to be done always thinking in the musical performance of the brass player. To enhance a complete understanding of the embouchure mechanism of a musician that suffered an orofacial trauma with direct implications on some important structures such as the teeth, the authors of this research recommend the usefulness of gathering professionals of different areas and expertise, namely, health care providers, engineers, and professional musicians that can bring added value to the final treatment by implementing a system for the monetarization of the sound quality such as the objective analysis of sound quality.

## 5. Conclusions

The present study provides detailed information regarding the sound quality of trumpet playing during musical performance taking in comparison different prosthetic solutions developed after the avulsion of two central incisors. This investigation supports the findings that the intra-oral appliance, which occupies less volume inside the oral cavity of the musician, is the best solution for the patient, not only in terms of comfort and aesthetic, but also in terms of the sound quality.

Decreasing pitch deviations have been found in the collected data with the HACI, followed by the TEATA, and finally the customized splint. Pitch deviations may result from the different intra-oral appliances due to the alteration of the mouth cavity, respectively the area occupied and modification/interaction with the anatomy, for example, the palate. Despite the lack of evidence or ground truth data on the possible association of these results with trumpet sound quality, we may affirm that the larger the mouth cavity area or, the smaller the appliance, the greater the degrees of control over sound formation by manipulating the embouchure by the lips, orofacial muscles, tongue, and teeth.

The necessity of an interdisciplinary approach for the treatment of an orofacial trauma in wind instrument players is an added value with the collaboration of different disciplines. The chance of having a multidisciplinary team that can implement these examinations, like the objective analysis of sound quality, promotes a compliance of the patient with the treatment plan. In addition to this, the musician understands there is an extra effort that goes beyond the clinical rehabilitation and has the challenge to understand how the evolution of his embouchure mechanism is in terms of sound.

Taking in consideration the example of this investigation, young wind instrument players should have dental impressions of their teeth made, so their dentist has the most reliable anatomy of the natural teeth in case of the occurrence of orofacial trauma. Likewise, the registration of their normal sound quality in terms of pitch, loudness, and timbre would allow clinicians and researchers to have the normal parameters for comparison in an eventual orofacial trauma.

## Figures and Tables

**Table 1:** List of score-independent audio descriptors adopted in our study as objective metrics for trumpet sound quality.

Category	Audio Descriptor	Definition
Pitch	Zero-Crossing Rate (ZCR)	Computes the zero-crossing rate of an audio time series, adopted as a rough indicator of pitch (i.e., fundamental frequency) for monophonic sounds
Loudness	Root mean square (RMS)	Computes the energy of an audio signal from its temporal (waveform) manifestation
	Root mean square of residual sound components (resRMS)	Computes the root mean square (i.e., energy) of the residual components of the sound, resulting from the median filtering method proposed by Fitzgerald, which suppresses stationary signals (i.e., the harmonic components) [41]
Timbre/Harmonic	Normalized spectral centroid (NSC)	Computes the center of gravity of the spectrogram, which is treated as a distribution over frequency bins. We further normalize the centroid value as the ratio to the fundamental frequency, resulting in a “unitless centroid” which allows the comparison of sounds with different fundamental frequencies while guaranteeing a robust perceptual indicator of sound brightness

**Table 2:** Global statistics of the collected descriptor data per intra-oral appliance. The following number denotes the intra-oral appliances: (1) HACI, (2) TEATA, and (3) Customized Splint.

Sound Attribute	Audio Descriptor	Intra-Oral Appliance	Mean	Median	IQR
Pitch	ZCR	(1)	0.1065	0.1011	0.0376
		(2)	0.1013	0.1030	0.0352
		(3)	0.1064	0.1064	0.0273
Loudness RMS		(1)	0.0962	0.0935	0.0273
		(2)	0.0934	0.0925	0.0470
		(3)	0.0966	0.0957	0.0529
resRMS		(1)	0.0035	0.00156	0.0018
		(2)	0.0028	0.00165	0.0014
		(3)	0.0033	0.00161	0.0013
Timbre NSC		(1)	7.9883	7.71546	3.8730
		(2)	7.9586	7.61702	4.0767
		(3)	8.1075	7.92492	3.0412

**Table 3:** Results from the non-parametric Friedman test of differences across repeated measures of the three intra-oral appliances conditions per descriptor. Register data is merged per appliance condition. Chi-square (χ^2^) and the statistical significance (*p*-value) are reported.

	Chi-Squared (χ^2^)	*p*-Value
ZCR	128.391	<0.001
RMS	8.221	0.0164
resRMS	5.695	0.058
NSC	715.529	<0.001
